# The effects of audio-visual perceptual characteristics on environmental health of pedestrian streets with traffic noise: A case study in Dalian, China

**DOI:** 10.3389/fpsyg.2023.1122639

**Published:** 2023-03-31

**Authors:** Xinxin Ren, Peng Wei, Qiran Wang, Wei Sun, Minmin Yuan, Shegang Shao, Dandan Zhu, Yishan Xue

**Affiliations:** ^1^School of Architecture and Fine Arts, Dalian University of Technology, Dalian, Liaoning, China; ^2^National Environmental Protection Engineering and Technology Center for Road Traffic Noise Control, Beijing, China; ^3^Research Institute of Highway Ministry of Transport, Beijing, China

**Keywords:** audio-visual environment, perception, traffic noise, pedestrian street, health evaluation, acoustic comfort

## Abstract

The COVID-19 pandemic has affected city dwellers’ physical and mental health and has raised concerns about the health of urban public spaces. This field investigation research in Dalian, China, examined the perceived audio-visual environment characteristics of urban pedestrian streets with traffic noise and their influences on the environmental health of the pedestrian streets. Five indicators reflecting psychological responses to environmental characteristics (*willingness to walk*, *relaxation*, *safety*, *beauty*, and *comprehensive comfort*) were used to measure environmental health of pedestrian streets with traffic noise. The results showed that *safety* was rated the highest, and *willingness to walk* was evaluated as the lowest among health evaluation indicators. The imageability and openness of the streetscape were associated with each health evaluation indicator. In contrast, the rhythm and continuity of the street buildings had a greater effect on *willingness to walk* than the other health indicators. There were negative correlations between *L*_Aeq_ for traffic noise and health evaluations. Positive health evaluations were observed when *L*_Aeq_ was less than 55 dBA. In contrast, soundscape indicators showed positive correlations with health evaluations, and acoustic comfort and noise annoyance, rather than sound preference and subjective loudness were associated with each health evaluation indicator. In terms of the combined audio-visual factors, acoustic comfort, the quantity of greening, annoyance, sky visibility, spatial scale, and building distance were examined as the determining factors affecting health evaluations, and 55.40% of the variance in health evaluations was explained by the soundscape and streetscape indicators. The findings provide references for better understanding the relationships between healthy experience and audio-visual perceptions. Moreover, they enable environmental health quality optimisation of pedestrian spaces considering audio-visual indicators and approaches in the post-epidemic era.

## 1. Introduction

The COVID-19 epidemic broke out in December 2019 and soon became a global problem, causing nearly 15 million deaths by 2021 (Grimley et al., 2022) directly or indirectly. Since the onset of the COVID-19 epidemic, public health has been severely affected (Giuntella et al., 2021; Pang et al., 2022); long periods of isolation and containment have prevented outdoor physical activity and caused a lack of social interaction, mental health challenges (Ma et al., 2022), heterogeneous effects on sleep (Ong et al., 2021), and increased depression (Ghebreyesus, 2020). These factors have highlighted the importance of outdoor activities on quality of life and raised concerns about the healthiness of urban open spaces. Streets are the basic organizational units of urban life and structure, and pedestrian spaces are an important part of urban public spaces for walking, rest, communication, and playing (Jacobs, 1993; Whyte, 1998; Ewing and Handy, 2009). However, since studies have shown that the COVID-19 typically spreads rapidly through droplets among high-density human populations (Jegan et al., 2021), the restriction of interpersonal interaction during the COVID-19 epidemic, in addition to people’s prevention awareness, could have changed street primary function into the dominant access space. The narrowed range of life activities meant that citizen travel gradually changed from using the urban public transport system to walking or cycling (Barbieri et al., 2021). For example, the Chinese government enforced strict regional gridded management to maximize safety, and to avoid commuting to work as far as possible (General Office of the National Health Commission of the People’s Republic of China, 2020), thereby enhancing access to pedestrian streets for citizen’s daily life. However, maintaining a safe social distance, personal hygiene protection and the improvement of immunity became a global consensus during the COVID-19 pandemic. Exercise, especially walking outside, can assist disease prevention and boost immunity (Ranasinghe et al., 2020; Oishi et al., 2021), thereby accelerating pedestrian streets as important urban open spaces for public health. Thus, the orientation of pedestrian streets in terms of healthiness could benefit the quality of city life and the construction of a healthy city (Rydin et al., 2012) in the post-epidemic era.

Most studies on the healthiness of urban streets have focused on the relationships between visual perceptual characteristics and healthy experience. Recently, Helbich et al. (2019) compared on-site street-view imagery with a satellite-derived street-view to assess green and blue spaces and their associations with the mental health of older adults. Lu et al. (2018) explored the relationship between street greening and walking behavior to promote physical activity. Koo et al. (2022) found that street-level factors were more strongly associated with walking-mode choice than neighborhood-level factors. Wang et al. (2019) focused on the visual sense of enclosures and found that street greening and buildings influenced walkability. Song et al. (2022) measured street greening at the line-of-sight level; they examined the relationship between street greening and walking satisfaction under the mediating effects of noise and PM 2.5 exposure. In addition, Zhao et al. (2020) found that increasing street plant visibility, reducing non-motor vehicles, and placing clear traffic signs on the streetscape can improve the psychological restoration of a street scene. Ewing et al. (2016) pointed out that street view features such as the proportion of street windows and frontage, the quantity of street furniture, and pedestrian traffic volume are important factors for walking and pedestrian-friendly environments. Izenberg and Fullilove (2016) investigated street contribution to community social cohesion, a condition of optimal health, and found that street wall permeability was a relevant element of street hospitality. Zhang et al. (2022) focused on the relationships between streetscape characteristics and mental health, and noted a significant positive correlation between residents’ mental health and the street enclosure. Many studies have highlighted walking behavior, street greening and diversified streetscape features as popular indicators of public health that are connected with pedestrian spaces. Few studies have focused on comprehensive healthy experiences of pedestrian streets based on people’s psychological responses to their perceived visual environment characteristics.

From an integrated audio-visual perception perspective, traffic noise—which can be widely distributed in pedestrian spaces—may cause psychological discomfort and thus influence the healthy experience of pedestrian streets (Ren et al., 2022a,b). Pedestrian streets adjacent to vehicular roads are particularly vulnerable to traffic noise. In 2011, the World Health Organization released a comprehensive report on the impact of noise on health (WHO, 2011), pointing out that noise pollution not only makes people uneasy but also causes or triggers many diseases, thereby reducing their life span. Thus, the pedestrian perception of acoustic environment quality is closely related to the healthy performance of a pedestrian street with traffic noise. Research on acoustic environment perception, such as soundscape research, has developed rapidly in recent years. Since the composer R Murray Schafer proposed using sound to build a healthy sound environment in the 1960s (Schafer, 1993), the soundscape concept has gradually been applied to sociology, esthetics, architecture, and other interdisciplinary research. In 2014, the International Organization for Standardization defined a soundscape as the sound environment perceived by individuals, groups, or communities in a given scene (International Organization for Standardization, 2014). Compared with traditional acoustic environment research focusing on physical phenomena, e.g., the sound pressure level (*SPL*), the soundscape is more concerned with perceptual constructs and the experience of the holistic acoustic environment (Yang and Kang, 2005). Diversified soundscape descriptors and indicators were used to express people’s perceptions of the acoustic environment in different contexts. For example, noise annoyance, pleasantness, perceived affective quality, restorativeness, and appropriateness have been suggested and used accordingly in previous studies (Aletta et al., 2018; International Organization for Standardization, 2018). For urban pedestrian streets, preference, loudness, communication, playfulness, and richness were used to characterize soundscaping urban shopping streets (Yu et al., 2016). Acoustic parameters and crowd density have been examined to predict the pleasantness and eventfulness of commercial streets (Hong and Jeon, 2020). The indicator of acoustic comfort was significantly associated with the assessment of pedestrian streets when sound levels of traffic noise were high (Hong and Jeon, 2013). Enclosure, continuity, and imaginability significantly influenced the acoustic comfort of residential streets dominated by traffic noise (Ren et al., 2022a,b). Although effective indicators have been identified to predict and improve pedestrians’ acoustic perception, there is still a dearth of material discussing how soundscape indicators could reflect on the healthy experience of pedestrians in urban street spaces. Thus, the soundscape indicator references on the health quality of pedestrian streets with traffic noise are limited.

**Figure 1:**
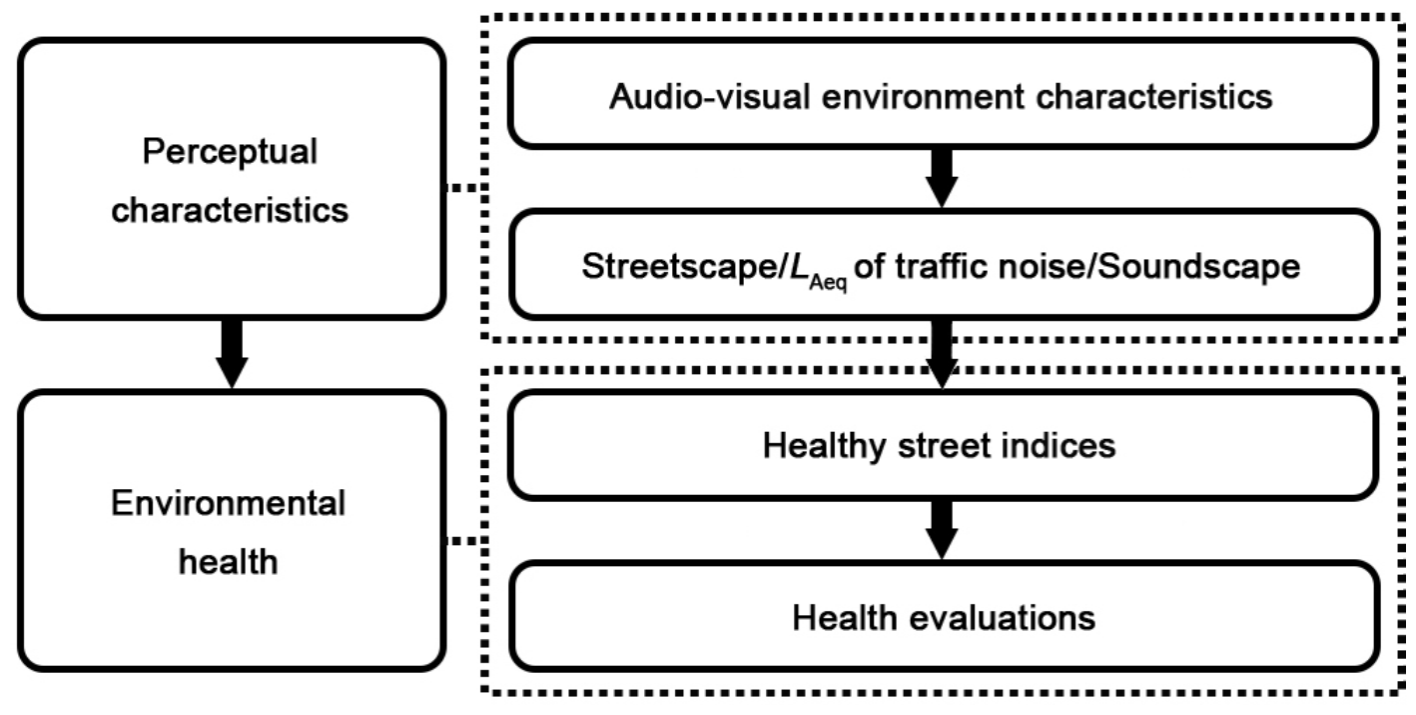
Hypothesized path model of audio-visual perceptions.

Therefore, this study mainly considers the audio-visual environment of pedestrian streets and follows a hypothesized path model (Figure 1). To provide empirical evidence of the health quality optimisation of pedestrian spaces considering audio-visual indicators and approaches in the post-epidemic era, we addressed the following research questions: How do (1) streetscape visual environment characteristics, (2) acoustic environment characteristics including *SPL* of traffic noise and soundscape quality, and (3) combined audio-visual characteristics when their perceptual indicators are both included, affect environmental health? To include relatively comprehensive aspects of environmental health, five indicators better reflecting psychological responses (*willingness to walk, relaxation*, *safety*, *beauty*, and *comprehensive comfort*) to environment characteristics were extracted from the Healthy Street Index (Healthy Streets, 2018) for measuring the health performance of existing pedestrian spaces. A field investigation with a questionnaire survey was adopted during the COVID−19 pandemic in Dalian, China, to obtain the on-site environmental and psychological reaction according to the real pedestrian street context.

## 2. Methodology

### 2.1. Case study area

**Figure 2:**
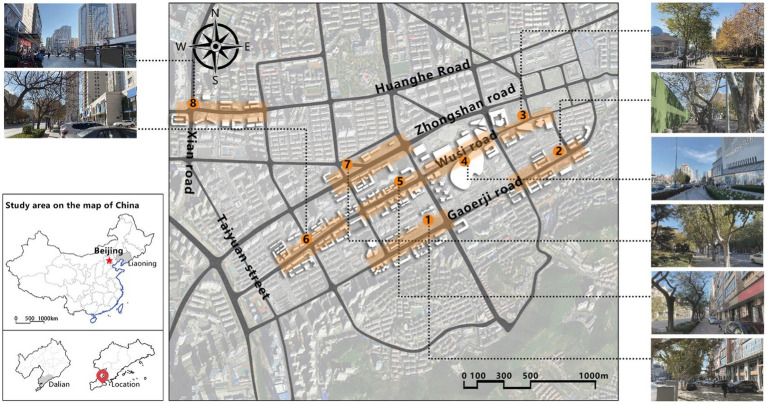
Study area.

With the acceleration of urbanization in China, the expansion of road networks has been proportional mainly to the scale of vehicular traffic. This exacerbates the extension and compression of vehicular roads into pedestrian spaces. Thus, pedestrian spaces adjacent to vehicular roads are inevitably influenced by traffic noise to varying degrees which has become typical of pedestrian routes. The city of Dalian is an important coastal center with a pleasant urban climate and a wide variety of public spaces, making it a typical liveable city in northeast China. A series of preventive measures to deter COVID-19 spread and its consequences have been implemented since the beginning of the epidemic in China (Bangura et al., 2020). Due to stringent preparative measures undertaken by Dalian, China, public events, schools and transportation have resumed and there were no new COVID-19 cases reported up until this study, which reduced risk levels for field investigation. As shown in Figure 2, the study area is located in the city’s central district, with flat topography and high pedestrian and vehicular traffic flow based on the highly accessible road network. Main city roads with a width of 10-30 m were in this district. These roads included Zhongshan Road (25 m), Huanghe Road (21 m), Gaoerji Road (15 m) and Wusi Road (15 m); the distribution of traffic sounds in the pedestrian space is representative of urban streets. In addition, this area is an old town in the city, with a mix of commercial and residential functions and a broad scope of demographic influences. The morphological characteristics and visual environment of pedestrian streets are typical for urban living streets. According to the diversity of street layouts, spatial patterns, and land use properties, eight pedestrian streets were selected for field investigation to obtain the audio-visual environment characteristics and health evaluations (Table 1).

**Table 1:** Case study site information.

Street plan	Street façade	Number of evaluations
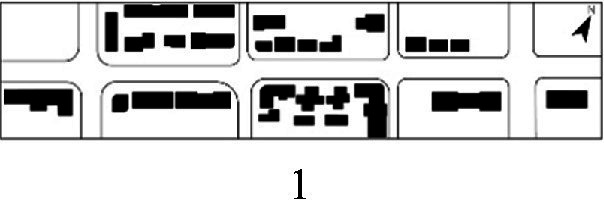	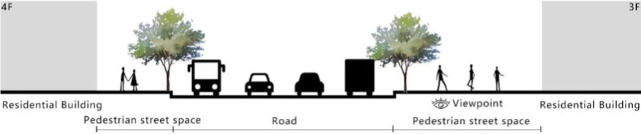	50
• Both sides of the street are residential areas, with public service facilities along the route. Low-height street buildings and wide sidewalks form the pedestrian space. The main functions in the space are neighbourhood communication, live entertainment, access, etc.		
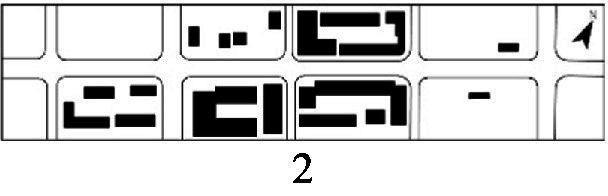	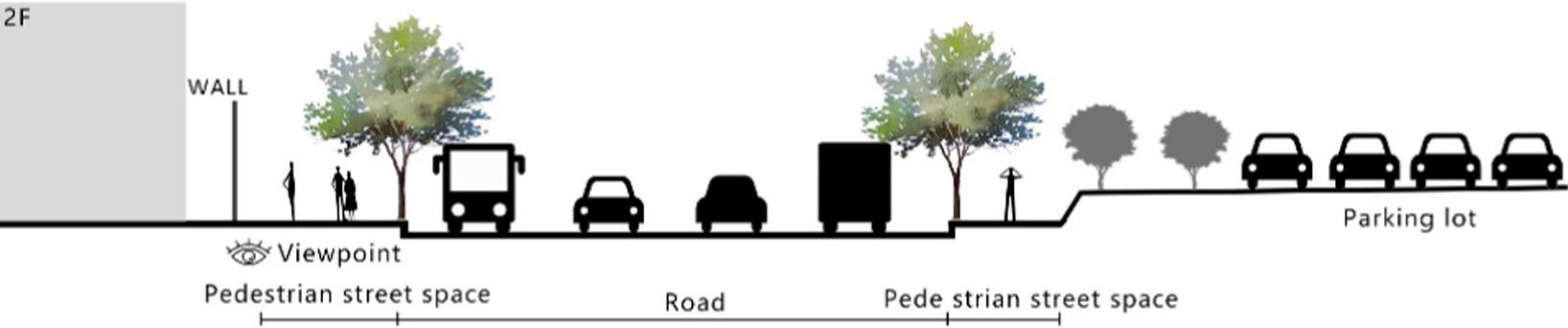	54
• Both sides of the street are residential areas. One side of the street is walled, and the other side is a parking lot. Low-height street buildings and narrow sidewalks mainly form the pedestrian space. The main activity in the space is access.		
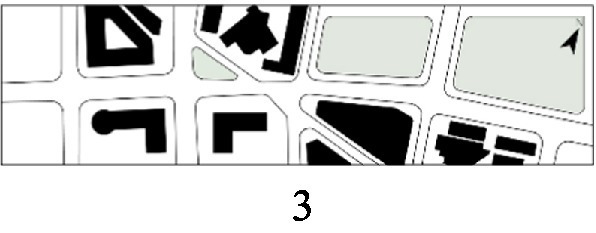	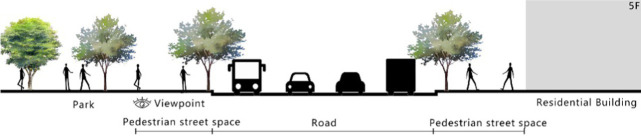	54
• One side of the street is dominated by leisure functions, with green areas and leisure spaces along the route. The pedestrian space is mainly formed by street greening and wide sidewalks. The main activities in the space are leisure activities, sports, access, etc.		
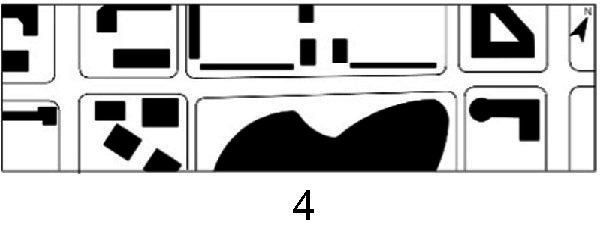	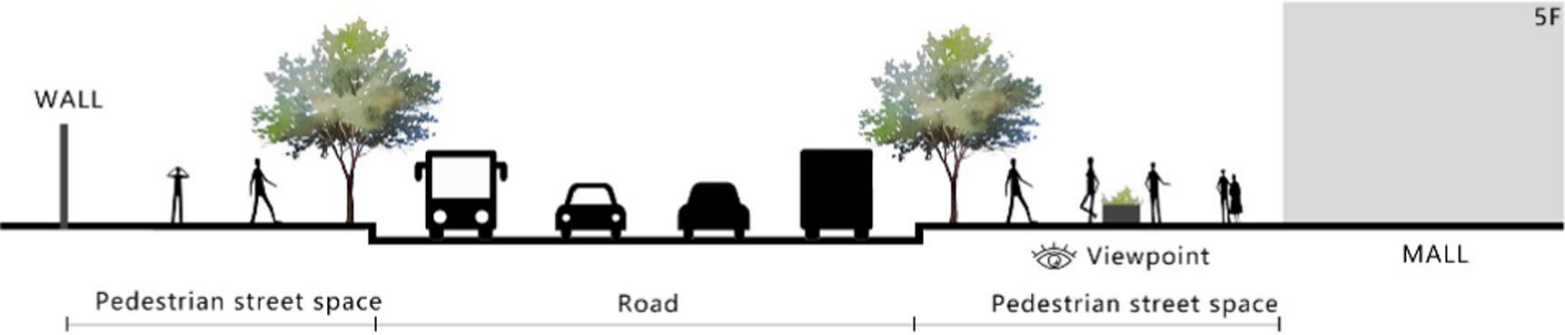	36
• Both sides of the street are commercial areas, with commercial buildings along the route. Low-height street buildings and wide sidewalks mainly form the pedestrian space. The main activities in the space are consumption, leisure entertainment, access, etc.		
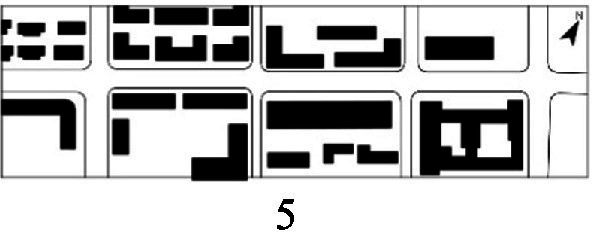	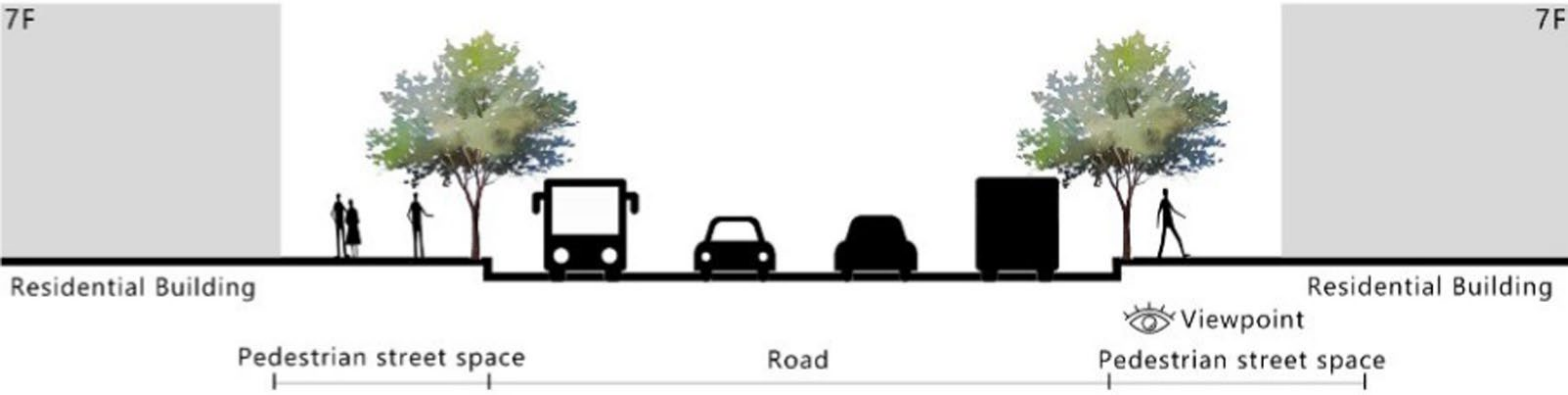	32
•Both sides of the street are residential areas, with public service facilities along the route. Medium-height street buildings and narrower sidewalks mainly form the pedestrian space. The main activities in the space are neighbourhood communication, access, etc.		
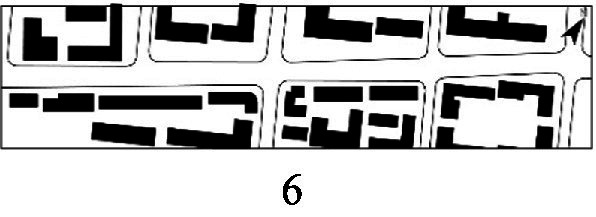	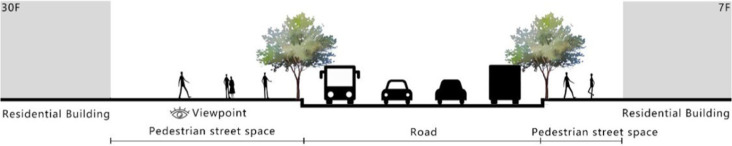	55
•Both sides of the street are residential areas, with public service facilities along the route. High street buildings and wide sidewalks mainly form the pedestrian space. The main activities in the space are neighbourhood communication, access, etc.		
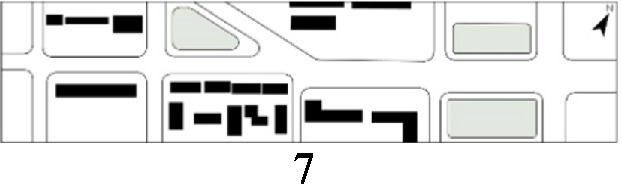	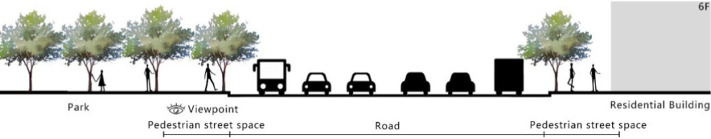	30
•One side of the street is dominated by leisure functions, with green areas and leisure spaces along the route. The pedestrian space is mainly formed by street greening and wide sidewalks. The main activities in the space are leisure activities, sports, access, etc.		
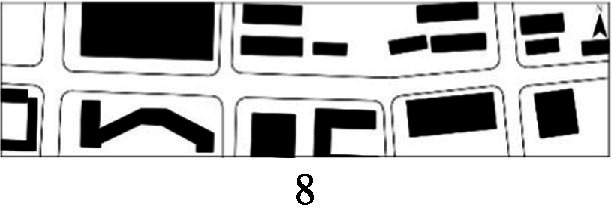	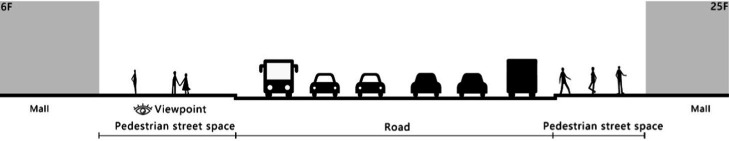	52
•Both sides of the street are commercial areas, with commercial buildings along the route. Medium-height street buildings and wide sidewalks mainly form the pedestrian space. The main activities in the space are consumption, leisure entertainment, access, etc.		

### 2.2. Acoustic environment measurement and simulation

The sound pressure level and traffic flow measurements were carried out on randomly selected work days and weekends in June and July 2022. Different time frames excluding rush-hour traffic, are included. A sound level meter was positioned at 1.5 m above the ground and 3.5 m away from the wall; no unique weather conditions occurred during the measurement (AM 9:00–11:00 and PM 15:00–17:00). The traffic flow and proportion of vehicle types were obtained and calculated using a video recorder. A total of 10 min of data were recorded at each measurement point. The distribution of traffic noise on pedestrian streets was simulated using SoundPLAN7.3 software. The RLS90 traffic noise prediction model (Deng et al., 2012) was adopted. The street and building data obtained from the field investigation and electronic map were used to set the road width, traffic flow, speed, and vehicle-type ratio in the software. The difference between the simulation and field-measured results was that the traffic sound that appeared in the real environment was within 0–1.5 dBA after calibration. The distribution of traffic noise in pedestrian spaces can be identified through traffic noise simulation. This enables the identification of the acoustic environment characteristics that are dominated by traffic, and the ability to exclude interference from other sound sources and events.

### 2.3. Questionnaire survey

A pre-survey was conducted with randomly selected residents walking in pedestrian spaces, who were informed of the research purpose and questionnaire items. During acoustic environment measurement at the measurement points, willing participants (who did not participate in the same questionnaire survey) were asked to complete a survey questionnaire on visual and audio environment characteristics and health evaluations based on their real perceptions after experiencing the on-site environment for 2–3 min (Kang, 2006). As shown in Table 2, the visual environment characteristics, including the streetscape features of intentionality, openness, continuity, and variability of the architectural interface (Ewing et al., 2016; Izenberg and Fullilove, 2016; Ren et al., 2022a), were measured on a five-point scale using the semantic differential method. As shown in Table 3, in addition to the *L*_Aeq_ of traffic noise, the acoustic environment characteristics were measured depending on typical soundscape indicators for pedestrian streets. These included acoustic comfort, subjective loudness, preference, and annoyance, which were expressed on a five-point scale ranging from ‘extremely not’ to ‘extremely so’ (International Organization for Standardization, 2018). For pedestrian street environmental health assessment, features of healthy streets were considered (Transport for London, 2018; Plowden, 2020). How participants experienced pedestrian spaces in terms of healthy urban street features was evaluated based on five indicators: *willingness to walk*, *relaxation*, *safety*, *beauty*, and *comprehensive comfort*. These indicators were extracted from corresponding ‘healthy street indices’, which were used for measuring existing pedestrian street health (Healthy Streets, 2018). More specifically, ‘*willingness to walk*’ was extracted from ‘*people choose to walk and cycle*’, ‘*relaxation*’ was connected to ‘*people feel relaxed*’, ‘*safety*’ corresponded to ‘*people feel safe*’, ‘*beauty*’ of the streetscape was associated with ‘*things to see*’, and ‘*comprehensive comfort*’ expressed pedestrian feelings toward the physical environment, service facilities and environment atmosphere (i. e., ‘*not too noisy*’, ‘*shade and shelter*’, ‘*everyone feels welcome*’). Because the five indicators were related to decisive dimensions for understanding pedestrian perceptions (Ferreira et al., 2022) and they can also directly describe environmental psychology when experiencing a real environment, single items were always used for each of them to characterize environmental quality in a number of previous studies (Xu et al., 2018; Jo and Jeon, 2021; Song et al., 2022; Ren et al., 2022a,b). Thus, single items with five-point scales were used to measure the health performance of the pedestrian streets in the questionnaire survey.

**Table 2:** Visual environment indicators for pedestrian streets based on semantic differential methods.

Indicators		Extremely so	A little	Neither/nor	A little	Extremely so	
Building height along the street	Low	−2	−1	0	1	2	High building height
Width of pedestrian space	Narrow	−2	−1	0	1	2	Wide pedestrian space
Sky visibility	Low	−2	−1	0	1	2	High sky visibility
Spatial scale	Closed	−2	−1	0	1	2	Opened in space scale
Interface height variation	Staggered	−2	−1	0	1	2	Flush building height
Interface concavity variation	Bumpy	−2	−1	0	1	2	Flat interface
Building distance along the street	Sparse	−2	−1	0	1	2	Continuous interface
Building form	Single	−2	−1	0	1	2	Extensive building forms
Quantity of street greening	Less	−2	−1	0	1	2	More street greening
Type of greenery	Single	−2	−1	0	1	2	Abundant greenery types
Facilities	Lack of	−2	−1	0	1	2	Full of service facilities
Cleanliness	Cluttered/Dirty	−2	−1	0	1	2	Tidy/Clean

**Table 3:** Soundscape and health evaluation indicators of pedestrian streets based on five-point scales.

	Indicator	Question	Answers
Soundscape	Acoustic comfort	How do you feel about the acoustic environment of this space, uncomfortable or comfortable?	1 = Uncomfortable, 2 = A little uncomfortable, 3 = Neither uncomfortable nor comfortable, 4 = A little comfortable, and 5 = Comfortable
	Subjective loudness	How do you feel about the acoustic environment of this space, noisy or quiet?	1 = Noisy, 2 = A little noisy, 3 = Neither noisy nor quiet, 4 = A little quiet, and 5 = Quiet
	Preference	How do you feel about the acoustic environment of this space, unpleasant or pleasant?	1 = Unpleasant, 2 = A little unpleasant, 3 = Neither unpleasant nor pleasant, 4 = A little pleasant, and 5 = Pleasant
	Annoyance	How do you feel about the acoustic environment of this space, annoying and not annoying?	1 = Annoying, 2 = A little annoying, 3 = Neither annoying nor non-annoying, 4 = A little not annoying, and 5 = Not annoying
Health evaluations	Willingness to walk	How would you be willing to walk on the pedestrian street according to your feeling about the environment?	1 = Extremely not, 2 = Not, 3 = Neither nor, 4 = A little, and 5 = Extremely so
	Relaxation	Do you feel relaxed on the pedestrian street according to your feeling about the environment?	
	Safety	Do you feel safe on the pedestrian street according to your feeling about the environment?	
	Beauty	Do you perceive the beauty of the streetscape according to your feeling about the environment?	
	Comprehensive comfort	How would you feel comprehensive comfort on the pedestrian street according to your feeling of the environment?	

A total of 395 interviewees participated in the survey, from which 363 valid questionnaires were obtained (the questionnaire was in Chinese). Each participant received a companion gift worth RMB 5–10 for completing the survey. According to the sampling information based on the survey, participants were from 18 to 60 years old, and young people were in the majority, with 26.45% of participants aged 20–29 years, and 41.05% aged 30–39 years. Middle-aged participants, aged 40–49 and 50–59 years made up 17.63 and 14.87%, respectively. Among all participants, 53.72% were male and 46.28% were female, a fairly balanced ratio.

### 2.4. Data analysis

To include various pedestrian space audio-visual characteristics, the questionnaires of the eight pedestrian streets were combined for analysis. The data from the questionnaire were calculated, and the Cronbach’s alpha coefficients for the three parts of visual environment perception, acoustic environment perception, and health evaluations were estimated to be 0.808, 0.805, and 0.855, respectively, which meet the requirements of good reliability (Ren et al., 2018a,b). The Kaiser-Meyer-Olkin (KMO) measures of sampling adequacy ≥0.70 with Bartlett’s spherical test results of *p* < 0.01 satisfied the validity of the questionnaire. The normality of each dimension was analyzed by a probability-probability (P–P) plot. The curve of normal P–P approximately follows a straight line, and an absolute difference of trend P–P less than 0.05, indicating that the variables follow a normal distribution (Field, 2013; Mishra et al., 2019). To explore the effects of audio-visual environment characteristics on the healthy performance of pedestrian streets, stepwise multiple linear regression analysis was used to reveal the significant streetscape or soundscape characteristics for each health evaluation indicator. Regression equations between *L*_Aeq_ and health evaluations were established through linear regression analysis after curve estimation. When audio-visual perceptual indicators were both considered, the main analysis was hierarchical multiple regression model which assessed the health evaluations influenced by the integrated audio-visual factors. The mean of health evaluations was set as the dependent variable and *SPL* of traffic noise, soundscape indicators of acoustic comfort, subjective loudness, preference and noise annoyance, and streetscape indicators referring to imaginability, openness, and architectural interface were set as predicator variables in groups. Finally, the effective factors were determined through significance tests for change in *R*^2^ to understand which the audio-visual variables accounted for the variance in health evaluations (Badri et al., 2021; Zhong et al., 2021).

## 3. Results

### 3.1. Characteristics of health evaluations on pedestrian streets with traffic noise

**Figure 3:**
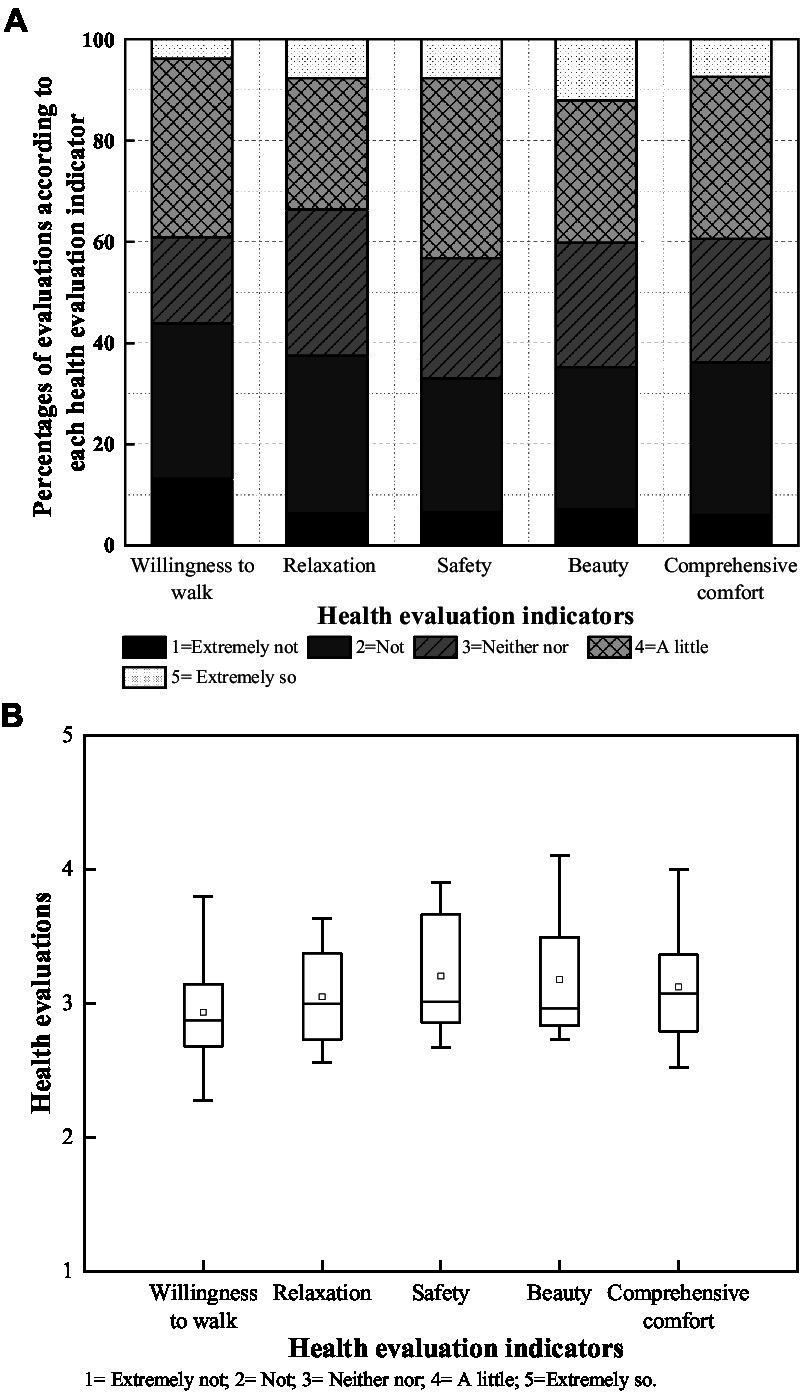
Pedestrian space characteristics regarding health evaluation indicators; box plots represent extremum and quantile health evaluations indicators. The circle symbols represent the means.

The health evaluations of pedestrian streets in terms of *willingness to walk*, *relaxation*, *safety*, *beauty* and *comprehensive comfort* are shown in Figure 3. As shown in Figure 3A, the percentage of evaluations of each health indicator differed between ‘extremely not’ (1 = ‘extremely not’) and ‘extremely so’ (5 = ‘extremely so’). However, less than one-half participants reported positive health evaluations according to different health evaluation indicators. Note that the positive evaluations (4, ‘a little’ and 5, ‘extremely so’) for *willingness to walk*, *relaxation*, *safety*, *beauty* and *comprehensive comfort* occupied 39.40, 34.17, 43.53, 40.22, and 39.67%, respectively. Figure 3B shows the distribution and mean of the evaluations. Note that *willingness to walk* had the most scattered evaluations with the lowest mean (2.93), *relaxation* had the most concentrated evaluations with a lower mean (3.05), and *safety* was connected to concentrated evaluations with the highest mean (3.20). The results indicate that the most fundamental safety needs were generally perceived higher in health evaluations than positive mental experiences, e.g., relaxation in mood and motivation in walking behavior. Among these, indicators with a greater distribution of evaluations could be influenced more by various perceived audio-visual factors based on the diversified streetscape features and traffic noise distribution of pedestrian streets.

### 3.2. Effects of visual environment characteristics on health evaluations of a pedestrian street

**Figure 4:**
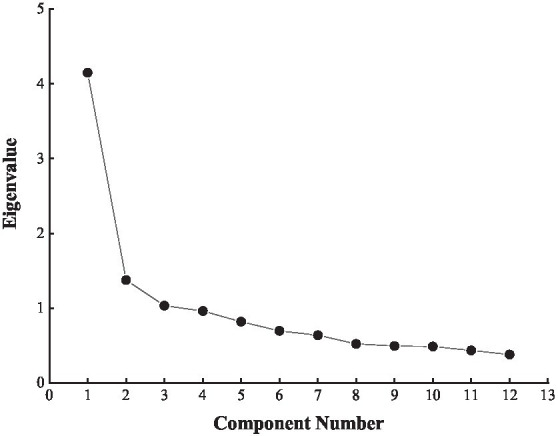
Scree plot.

A principal component analysis with varimax rotation was conducted to characterize the perceived visual environment of the pedestrian street. With a criterion factor of eigenvalue >1, four principal components were explored, and eigenvalues of the first three principal components were greater than the criteria factor of 1, and the fourth was close to 1 (0.96, Figure 4). As shown in Table 4, the four principal components were ordered according to factor loading. Component 1 (20.99%) could be summarized as the imageability of the streetscape, including building form, quantity of street greening, diversity of street vegetation, service facilities, and cleanliness. Component 2 (18.09%) was associated with openness, including the sidewalk width, sky visibility, and space scale. Component 3 (12.73%) represented differences in the street interface regarding height and concavity, which can be perceived as the rhythm of the street architecture. Component 4 (10.76%) included the building height and interval space between the buildings, namely, the continuity of the street buildings. Based on the visual perception of the four aspects and the evaluations of semantic differential methods, the characteristics of imageability were clearly presented: simple building forms, lack of service facilities, and abundant street greening. It is noted that the 4 principal components explained 62.57% of the total variance of the visually perceived environment, which is higher than in previous studies that explored perceptual acoustic environment characteristics through semantic processing (58–61%) (Kang and Zhang, 2010; Ren, 2023). But the percentage of unexplained variability could include detailed design and landscape attitudes of the streetscape, such as building color, visual esthetic quality, and landscape preference, all playing a role in visual landscape perception (Ren, 2019; Ren et al., 2022b).

**Table 4:** Principal component analysis for visual environment characteristics.

Classification	Visual environment characteristics	Factors				Evaluations
		1 (20.99%)	2 (18.09%)	3 (12.73%)	4 (10.76%)	
Imageability	Building form	0.53				−0.17
	Quantity of street greening	0.64				0.13
	Type of greenery	0.75				0.10
	Facilities	0.72				−0.24
	Cleanliness	0.65				−0.04
Openness	Width of pedestrian space		0.70			0.03
	Sky visibility		0.79			0.00
	Spatial scale		0.67			0.10
Rhythm of street buildings	Interface height variation			0.71		−0.02
	Interface concavity variation			0.83		−0.06
Continuity of street buildings	Building height along the street				0.84	−0.23
	Building distance along the street				0.70	−0.33

Kaiser-Meyer-Olkin measure of sampling adequacy: 0.855 (*p* = 0.000); cumulative %: 62.67; extraction method: principal component analysis; rotation method: varimax with Kaiser normalization; *N* = 363.

Correspondingly, the relationships between visual environment characteristics and health evaluations were described using stepwise multiple linear regression with health evaluation indicators as dependent variables and visual environment characteristics as independent variables. There were positive correlations between perceived visual environment characteristics and health evaluations (Table 5). Visual factors explained the highest degree of *willingness to walk* (*R*^2^(adj) = 0.320, *p* < 0.01) and the lowest degree of *relaxation* (*R*^2^(adj) = 0.232, *p* < 0.01). More specifically, imageability and openness were significantly related to each health evaluation indicator. In particular, the quantity of street greening and sky visibility significantly affected all *willingness to walk*, *relaxation*, *safety*, *beauty*, and *comprehensive comfort* indicators. Whereas the effectiveness of other factors in the aspects of imageability and openness were determined by different health evaluation indicators. Building form, cleanliness, type of greenery, facilities had significant influences on *willingness to walk*, *safety*, *beauty*, and *comprehensive comfort*, respectively. Spatial scale had significant influences on *relaxation*, *beauty*, and *comprehensive comfort*, but had an insignificant influence on the other health evaluation indicators. Comparatively, the effects of rhythm and continuity of street buildings on health evaluations were weaker than those of imageability and openness. Notably, rhythm and continuity of street buildings both had significant influences on *willingness to walk* and *relaxation*; in particular, more visual factors such as interface concavity variation, building height and building distance were associated with *willingness to walk*. In terms of continuity of street buildings, building height and building distance were significant factors affecting *safety* and *comprehensive comfort*, and in terms of rhythm of street buildings, interface concavity variation was a key factor for *beauty*.

**Table 5:** Significant factors of visual environment characteristics for health evaluations from the stepwise multiple linear regression analysis.

Dependent variable	Variable classification	Variables	Unstandardized Beta	Standardized Beta	*t*	Sig.	Tolerance	VIF
Willingness to walk *R*^2^ = 0.332, *R*^2^(adj) = 0.320, *p* ≤ 0.01.	Imageability	Quantity of street greening	0.202	0.198	3.929	0.000	0.738	1.356
		Building form	0.099	0.096	2.019	0.044	0.826	1.211
	Openness	Sky visibility	0.244	0.236	4.734	0.000	0.753	1.328
	Rhythm of street buildings	Interface concavity variation	0.238	0.221	4.880	0.000	0.918	1.089
	Continuity of street buildings	Building height	0.140	0.133	2.955	0.003	0.924	1.082
		Building distance	0.088	0.094	2.014	0.045	0.867	1.153
Relaxation *R*^2^ = 0.242, *R*^2^(adj) = 0.232, *p* ≤ 0.01.	Imageability	Quantity of street greening	0.152	0.160	2.969	0.003	0.733	1.364
	Openness	Sky visibility	0.142	0.148	2.610	0.009	0.660	1.514
		Spatial scale	0.135	0.144	2.550	0.011	0.662	1.511
	Rhythm of street buildings	Interface concavity variation	0.209	0.209	4.262	0.000	0.886	1.128
	Continuity of street buildings	Building distance	0.089	0.102	2.167	0.031	0.959	1.042
Safety *R*^2^ = 0.259, *R*^2^(adj) = 0.249, *p* ≤ 0.01.	Imageability	Quantity of street greening	0.188	0.195	3.564	0.000	0.696	1.437
		Cleanliness	0.146	0.150	2.862	0.004	0.760	1.316
	Openness	Sky visibility	0.111	0.114	2.089	0.037	0.697	1.435
	Continuity of street buildings	Building height	0.178	0.179	3.514	0.000	0.795	1.257
		Building distance	0.133	0.150	3.147	0.002	0.918	1.089
Beauty *R*^2^ = 0.287, *R*^2^(adj) = 0.277, *p* ≤ 0.01.	Imageability	Quantity of street greening	0.199	0.194	3.409	0.001	0.616	1.624
		Type of greenery	0.199	0.201	3.607	0.000	0.642	1.557
	Openness	Sky visibility	0.117	0.113	2.035	0.043	0.648	1.543
		Spatial scale	0.127	0.127	2.286	0.023	0.648	1.543
	Rhythm of street buildings	Interface concavity variation	0.116	0.108	2.294	0.022	0.904	1.106
Comprehensive comfort *R*^2^ = 0.286, *R*^2^(adj) = 0.276, *p* ≤ 0.01.	Imageability	Quantity of street greening	0.170	0.178	3.258	0.001	0.673	1.485
		Facilities	0.134	0.142	2.896	0.004	0.829	1.206
	Openness	Sky visibility	0.136	0.140	2.497	0.013	0.634	1.577
		Spatial scale	0.161	0.172	3.133	0.002	0.663	1.508
	Continuity of street buildings	Building height	0.138	0.140	2.863	0.004	0.831	1.204

### 3.3. Effects of acoustic environment characteristics on health evaluations of a pedestrian street

Table 6 shows *SPL* of traffic noise at different time frames according to the measurement points. As noise maps of the pedestrian streets were also shown, such that the traffic noise distributed in the pedestrian spaces can be observed. It was found that although noise levels decreased with increasing distance from the road, the majority of pedestrian spaces were affected by traffic noise above 65 *L*_Aeq_. A high *L*_Aeq_ above 70 dBA was concentrated on the road intersection and the surrounding area. A relatively lower *L*_Aeq_ was distributed along the pedestrian space with a continuous building interface. Generally speaking, the mean *L*_Aeq_ was 65.82 and 66.43 dBA for the morning (9:00–11:00) and the afternoon (15:00–17:00), respectively; thus, the traffic noise levels could be relatively stable at high levels depending on the times of outside activities (9:00–11:00/15:00–17:00).

**Table 6:** *L*_Aeq_ of the pedestrian space according to the measurement points at different time frames, where the noise maps of pedestrian streets 1–8 (AM 9:00–11:00) were also shown.

Time frames	*SPL* of traffic noise/Street numbers								
		1	2	3	4	5	6	7	8
AM 9–11:00	Mean	67.69	71.23	62.88	63.86	66.22	64.16	67.33	63.17
	SD	2.88	3.51	2.16	2.06	2.16	2.93	4.32	2.92
PM 15–17:00	Mean	66.05	70.95	65.43	65.32	65.48	65.05	67.26	65.91
	SD	3.06	3.70	2.03	1.36	2.18	2.85	4.46	3.75
AM 9–11:00	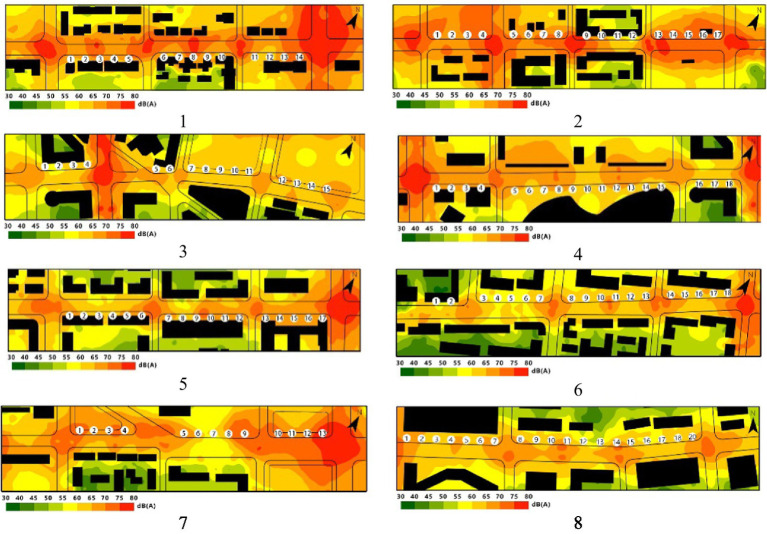								

**Figure 5:**
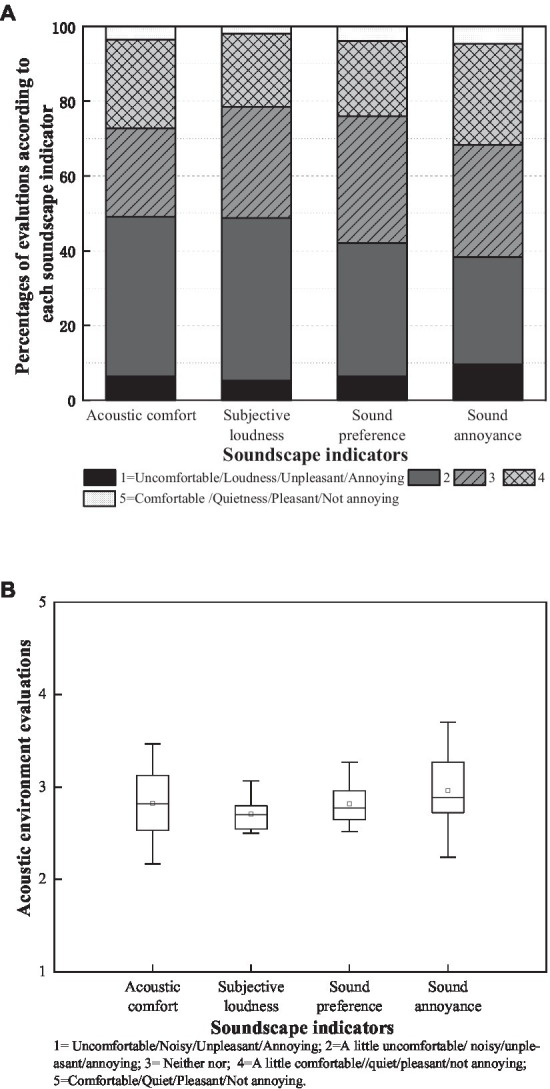
Acoustic environment characteristics regarding soundscape indicators; box plots represent the extremum and quantile soundscape evaluations. The circle symbols represent the means.

The soundscape evaluations of pedestrian streets are shown in Figure 5. In Figure 5A, the positive evaluations (4, ‘a little’ and 5, ‘extremely so’) for acoustic comfort, subjective loudness, preference and annoyance occupied 27.27, 21.49, 23.97 and 31.68%, respectively. Figure 5B shows the distribution and mean of the soundscape indicator evaluations. Note that all evaluations were slightly lower than the median (2.75–2.88). Acoustic comfort and annoyance had more scattered evaluations than subjective loudness and preference. These ranged from ‘a little uncomfortable’ (2.17) to ‘neither uncomfortable nor comfortable’ (3.47) and ‘a little annoyance’ (2.24) to ‘a little not annoying’ (3.70). The results indicated that improving the indicator evaluations of acoustic comfort and annoyance could benefit positive auditory perception. Notably, although high traffic noise levels were distributed in the pedestrian streets, soundscape indicator evaluation means were not extremely low, even with some positive ratings. This may be partly because people are accustomed to the typical pedestrian spaces adjacent to vehicular roads and become insensitive to the noisy environment, and/or partly due to relatively low traffic noise at the time of the acoustic environment evaluation.

Table 7 presents the relationships between acoustic environment characteristics (soundscape indicators) and health evaluations based on the stepwise multiple linear regression. As expected, significant correlations were found between soundscape indicators and health evaluations. Soundscape indicators explained the highest degree of *willingness to walk* (*R*^2^(adj) = 0.397, *p* < 0.01) and the lowest degree of *beauty* (*R*^2^(adj) = 0.155, *p* < 0.01). For the effects of soundscape on health evaluation indicators, acoustic comfort and noise annoyance rather than sound preference and subjective loudness were significantly associated with all *willingness to walk*, *relaxation*, *safety*, *beauty*, and *comprehensive comfort* indicators. This means that the positive perception of acoustic comfort and noise annoyance could be the most important consideration for pedestrian street health quality, particularly for promoting healthy walking behavior (*willingness to walk*). Additionally, sound preference was another significant and effective factor for *safety* and *beauty*, besides acoustic comfort and noise annoyance. This result suggests that although fewer healthy evaluations in perceived *safety* and *beauty* were explained by soundscape indicators, multidimensional soundscape quality could be taken into account for improving pedestrian street healthy features.

**Table 7:** Significant factors of soundscape indicators for health evaluations from the stepwise multiple linear regression analysis.

Dependent variable	Variables	Unstandardized Beta	Standardized Beta	*t*	Sig.	Tolerance	VIF
Willingness to walk *R*^2^ *= 0.401, R*^2^*(adj) = 0.397,* *p ≤ 0.01*	Acoustic comfort	0.595	0.519	10.195	0.000	0.642	1.558
	Annoyance	0.181	0.160	3.265	0.001	0.642	1.558
Relaxation *R*^2^ = 0.277, *R*^2^(adj) = 0.273, *p* ≤ 0.01	Acoustic comfort	0.460	0.431	7.698	0.000	0.642	1.558
	Annoyance	0.141	0.139	2.489	0.013	0.642	1.558
Safety *R*^2^ = 0.236, *R*^2^(adj) = 0.230, *p* ≤ 0.01	Acoustic comfort	0.317	0.293	4.929	0.000	0.604	1.656
	Annoyance	0.174	0.169	2.775	0.006	0.575	1.740
	Preference	0.127	0.112	2.026	0.043	0.695	1.439
Beauty *R*^2^ = 0.162, *R*^2^(adj) = 0.155, *p* ≤ 0.01	Annoyance	0.192	0.176	2.764	0.006	0.575	1.740
	Preference	0.190	0.158	2.720	0.007	0.695	1.439
	Acoustic comfort	0.174	0.151	2.435	0.015	0.604	1.656
Comprehensive comfort *R*^2^ = 0.365, *R*^2^(adj) = 0.362, *p* ≤ 0.01	Acoustic comfort	0.378	0.352	6.724	0.000	0.642	1.558
	Annoyance	0.330	0.324	6.178	0.000	0.642	1.558

**Figure 6:**
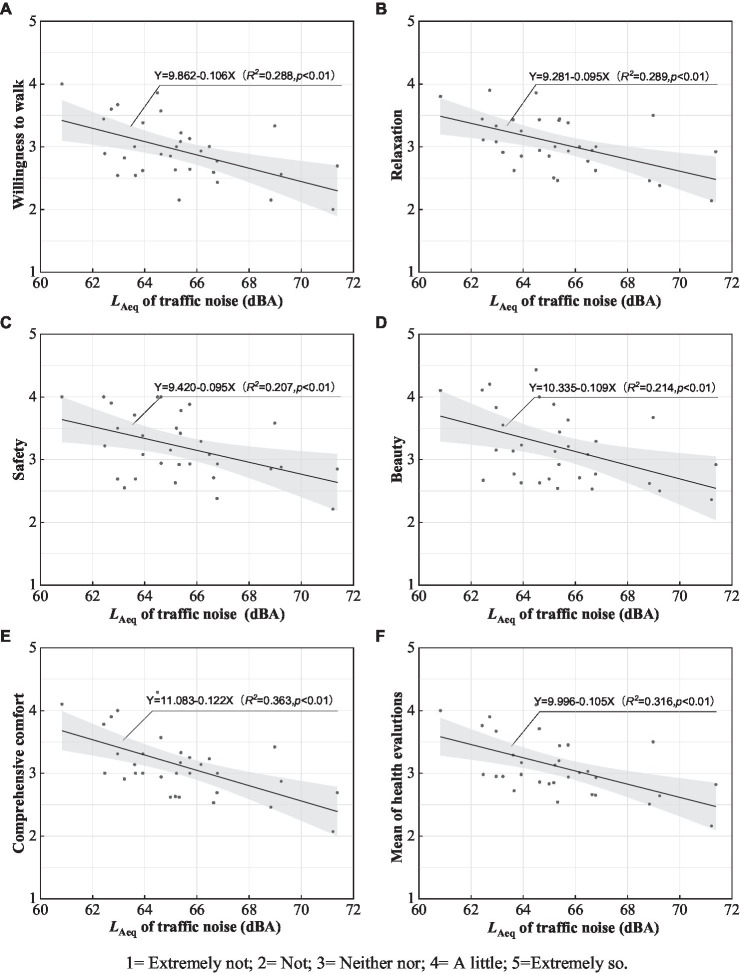
Relationships between *L*_Aeq_ of traffic noise and healthy evaluations of pedestrian streets; gray areas represent 95% confidence intervals of the evaluations.

Although soundscape indicators were found to effective in affecting health evaluations, the *L_Aeq_* of traffic noise is a more controllable indicator using a quantitative approach. To discuss the ideal threshold of traffic noise for healthy experiences on pedestrian streets, linear regression analysis was conducted to express the relationships between *L_Aeq_* of traffic noise and health evaluations (the Linear model had the best fit with a higher correlation coefficient based on the results of curve estimation when explaining interactions between *L*_Aeq_ and health evaluations). According to combined data from the *L*_Aeq_ traffic noise database and questionnaires, the relationships between *L*_Aeq_ of traffic noise and health evaluations are presented in Figure 6. *L*_Aeq_ of traffic noise as independent variables were used to predict each of the five health evaluation indicators and health evaluation means, respectively. Generally, health evaluation indicators exhibited a negative linear trend over the effects of *L*_Aeq_, with *R*^2^ = 0.207–0.363 and *p* < 0.01. The relationships can be best described by linear equations: *Y*_*Willingness to walk*_ = 9.862–0.106*X*_*LAeq*_, *Y_Relaxation_* = 9.281–0.106*X*_*LAeq*_, *Y_Safey_* = 9.420–0.095*X*_*LAeq*_, *Y_Beauty_* = 10.335–0.109*X*_*LAeq*_, *Y_Comprehensive comfort_* = 11.083–0.122*X*_*LAeq*_, and *Y_Mean of health evaluations_* = 9.996–0.105*X*_*LAeq*_. As the health evaluations increased from 4 = ‘a little’ to 5 = ‘extremely so’, traffic noise thresholds for *willingness to walk*, *relaxation*, *safety*, *beauty* and *comprehensive comfort* were 55.30 ~ 45.87 dBA, 55.59 ~ 45.06 dBA, 57.05 ~ 46.53 dBA, 58.12 ~ 48.94 dBA and 58.06 ~ 49.86 dBA, respectively. A more strict *L*_Aeq_ of 57.10 ~ 47.58 dBA was examined for the mean values of the indicator evaluations. The results indicate that although the reasonable *L*_Aeq_ differed according to different psychological experiences based on the health indicators, controlling traffic noise at levels lower than 55 dBA is necessary for positive health evaluations on pedestrian streets. However, a lower *L*_Aeq_ is difficult to achieve on pedestrian walkways near busy roads using noise control alone. Space construction and soundscape optimisation in terms of acoustic environment characteristics should utilize an integrated approach to improve the healthy performance of pedestrian spaces.

### 3.4. Effects of audio-visual environment factors on the health evaluation of pedestrian street

A hierarchical multiple regression model was established to analyze the comprehensive influence of audio-visual factors on the health evaluations of pedestrian streets; the mean values of health evaluations were dependent variables, and audio-visual factors were independent variables. The covariance test of the regression model showed that the variance inflation factor (VIF) was less than 10, and the Durbin–Watson value was 1.74. This indicates that no collinearity occurred and that the model could be effective for construction. More specifically, in Table 8, Models 1 and 2 examined the health evaluations gradually influenced by auditory factors. Soundscape indicators of acoustic comfort and noise annoyance (*p* < 0.01) were more effective than *SPL* of traffic noise (*L*_Aeq_) (*p* > 0.05) in predicting health evaluations. As Model 3 reveals, adding the imageability independent variable increased *R*^2^(adj) from 0.425 to 0.511 (*p* < 0.01). This result indicates that imageability (quantity of street greening and cleanliness) significantly affected health evaluations and soundscape indicators. Correspondingly, adding the openness independent variable increased *R*^2^(adj) from 0.511 to 0.538 (*p* < 0.01) in Model 4. Notably, sky visibility and spatial scale became significant visual factors affecting health evaluations as opposed to cleanliness. As the interface of street buildings was included in Model 5, a small increase in *R*^2^(adj) from 0.538 to 0.554 (*p* < 0.01) was observed. Although the added variable was less significant in environmental health compared with the other determinants, the effective factor of building distance was explored for the final model. It indicates that when the audio-visual factors of soundscape, imageability, openness, and architectural interface were connected, a higher amount of variance in health evaluations was explained based on *R*^2^(adj) = 0.554 (*p* < 0.01). It can be seen that six decisive audio-visual factors were identified in descending order for the degree of influence: acoustic comfort (*β* = 0.294), the quantity of greening (*β* = 0.123), noise annoyance (*β* = 0.113), sky visibility (*β* = 0.082), spatial scale (*β* = 0.072), and building distance (*β* = 0.057).

**Table 8:** Summary of hierarchical multiple regression analysis of the effects of audio-visual factors on health evaluations of a pedestrian street.

		Model 1	Model 2	Model 3	Model 4	Model 5
*SPL* of traffic noise	*L*_Aeq_	−0.028	−0.0001	0.004	0.006	0.010
Soundscape indicators	Acoustic comfort		0.379**	0.324**	0.314**	0.294**
	Subjective loudness		−0.004	0.020	0.015	0.017
	Preference		0.092	0.028	0.006	−0.005
	Annoyance		0.195**	0.123**	0.120**	0.113**
Imaginability	Building form			0.058	0.029	0.014
	Quantity of street greening			0.168**	0.124**	0.123**
	Type of greenery			0.033	0.011	0.019
	Facilities			−0.005	−0.006	−0.011
	Cleanliness			0.092**	0.057	0.043
Openness	Width of pedestrian space				0.039	0.041
	Sky visibility				0.088*	0.082*
	Spatial scale				0.088*	0.072*
Architectural interface	Interface height variation					0.047
	Interface concavity variation					0.059
	Building height					0.022
	Building distance					0.057*
	*R* ^2^	0.006	0.433	0.525	0.555	0.575
	*R*^2^(adj)	0.004	0.425	0.511	0.538	0.554
	*ΔR^2^*	0.006	0.426	0.092	0.030	0.020
	*ΔF*	2.360	67.122	13.586	7.830	4.148
	*Sig*.	0.125	0.000**	0.000**	0.000**	0.003**

^**^p < 0.01; ^*^p < 0.05.

## 4. Discussion

In this study, perceived factors in terms of the visual environment were found to significantly impact pedestrians’ health experiences while walking (Table 8). Active walking can improve the physical health of residents. Previous studies have indicated that factors such as sidewalk width, street greenery, and facilities impact walking behavior (Saelens and Handy, 2008; Cao et al., 2009; van Dillen et al., 2012; Kim et al., 2014; Ferrer et al., 2015). Street greening and wide walking spaces can evoke cheerful moods, while facilities such as lighting have a greater impact on safety (Kelly et al., 2011). Based on field investigation during the COVID−19 pandemic in Dalian, China, the health evaluations of pedestrian streets in terms of *willingness to walk*, *relaxation*, *safety*, *beauty* and *comprehensive comfort* were somewhat unsatisfactory (Figure 3). In particular, the evaluations of *willingness to walk* were the lowest, whereas those of *safety* were slightly higher. However, positive perceptions of streetscape imageability and spatial openness contributed to the health evaluations of pedestrian streets to a greater extent, with significant influences on each health evaluation indicator. The architectural interface characteristics, such as rhythm and continuity of street buildings, also played a significant role in the health evaluations, especially for *willingness to walk* (Table 5). In addition, it is consistent with previous studies’ emphasis on the role of greenery in physical and mental health (Lu et al., 2018; Helbich et al., 2019; Ren, 2019; Wang et al., 2019; Koo et al., 2022; Song et al., 2022). The importance of street greening for every health evaluation indicator was highlighted in this study (Table 5).

Visual environment factors could be the primary considerations for health evaluations of pedestrian streets based on previous studies (Rehan, 2016); furthermore, the acoustic environmental quality of pedestrian streets due to traffic noise has a notable impact. The latest report on environmental noise pollution in China states that the *L*_day_ in 2021 was 66.6 dBA (Ministry of Ecology and Environment of the People’s Republic of China, 2021). While still within the *L*_Aeq_ noise limits, health risks still exist based on auditory sensors (Hu, 2012). In this study, traffic sound levels distributed in pedestrian spaces generally exceeded 65 dBA and were negatively correlated with health evaluations. Concerning positive health evaluations, the ideal thresholds of the sound levels were discussed (Figure 6), and the effects of soundscape indicators were analyzed simultaneously. For example, acoustic comfort and noise annoyance were positively correlated with each of the health evaluation indicators (Table 7). In other words, the potential of soundscape indicators to promote the healthy performance of pedestrian streets was revealed. A variety of audio-visual factors may have an integrated impact on the process of subjective evaluations (Ren and Kang, 2015a,b; Ren et al., 2018a,b). The combined effects of audio-visual factors—acoustic comfort, the quantity of greening, noise annoyance, sky visibility, spatial scale, and building distance—were the key determinants, covering a high percentage of the variance for health evaluations in pedestrian spaces with traffic noise (Table 8). As opposed to traditional methods, i.e., controlling *L*_Aeq_ and measuring noise annoyance for pedestrian spaces, a collaborative consideration of acoustic comfort indicators that promotes positive psychological experiences should be assessed. Moreover, improving environmental quality planning and renewal measures such as increasing street greening, improving openness, continuous interface and better soundscape design should improve user psychological or behavioral feedback and incentives. Our findings also explain a substantial amount of variance in health evaluations influenced by audio-visual environment characteristics (55.40%). However, interactions among visual environment cues (e.g., quantity of street greening, sky visibility, spatial scale, and building distance), acoustic comfort, and noise annoyance may affect the healthy experience in specific ways. Hence, limited aspects not covered in depth in this study should be pursued in future research through specific scenario analysis and laboratory studies to identify further empirical references for the relationship between audio-visual interaction and street health environment optimisation. Secondly, because the subjective response of soundscape quality is driven primarily by the dominant sounds (Pérez-Martínez et al., 2018; Ren, 2023), traffic noise and its *SPL* were calculated when pedestrian spaces adjacent to vehicular roads were considered in this study. However, footsteps and human speech, could be heard when traffic noise was lower in the real environment. The interactions between a series of sounds and composite *SPL* make environmental quality research more difficult and complicate the traffic noise control. It was never the aim of this study, but this was an interesting and beneficial topic. Third, there are other perceived physical environment factors other than audio-visual perceptual cues affecting environmental health, such as urban street air pollution. Therefore, studies which include the effects of multidimensional characteristics on environmental health are recommended in the future.

## 5. Conclusion

This study examined city dwellers’ environmental psychology responses to environmental health of pedestrian streets. The effects of perceived audio-visual environment characteristics on environmental health were analyzed and discussed. Particular attention has been paid to people’s mental experiences on pedestrian streets after several waves of COVID-19 in Dalian, China. A field investigation of audio-visual environment perceptions, traffic noise simulation, and health evaluations of typical pedestrian spaces with traffic noise was conducted. The primary aim was to collect data on streetscapes, *L*_Aeq_ of traffic noise, and soundscape, and conduct health evaluations of *willingness to walk*, *relaxation*, *safety*, *beauty* and *comprehensive comfort*. The results of this case study are summarized.

1. The health evaluations of pedestrian streets regarding *willingness to walk*, *relaxation*, *safety*, *beauty,* and *comprehensive comfort* were somewhat unsatisfactory. Approximately 40% of evaluations were positive for each health evaluation indicator. In particular, the mean evaluation of *willingness to walk* was the lowest (2.93), and *safety* was the highest (3.20), based on a five-point scale.
2. Four main factors were explored to characterize the perceived visual environment: imageability, openness, rhythm, and continuity of street buildings. Imageability and openness of the streetscape were significantly associated with each health evaluation indicator. The rhythm and continuity of the street buildings were more effective on *willingness to walk* and *relaxation* than the other health indicators.
3. *L*_Aeq_ of traffic noise and soundscape indicators were used to characterise the perceived acoustic environment. Negative relationships were confirmed between *L*_Aeq_ and health evaluations, and positive health evaluations were observed where *L_Aeq_* was lower than 55 dBA. Positive relationships were identified between soundscape indicators and health evaluations. Acoustic comfort and noise annoyance rather than sound preference and subjective loudness were significantly associated with each health evaluation indicator. Sound preference was another statistically effective indicator for *safety* and *beauty*.
4. When the audio-visual factors of *L*_Aeq_ of traffic noise, soundscape and imageability, openness, and architectural interface of streetscape were connected, a higher variance in health evaluations was explained based on *R*^2^(adj) = 0.554 (*p* < 0.01). Acoustic comfort, the quantity of greening, noise annoyance, sky visibility, spatial scale, and building distance were examined for the degree of influence as the determining factors in descending order.

Overall, the present study provides empirical evidence on how environmental perceptions are based on visual and audio cues that benefit environmental psychology responses and audio-visual environmental quality, focusing on the healthy experience of pedestrians in the post-epidemic era. In addition, the presented findings support the use of audio-visual indicators to optimize the environmental health of pedestrian spaces with traffic noise in terms of mental experience. Moreover, the study suggests that different practical strategies could be considered for traffic noise control, space layout, and soundscape planning, designing, and optimisation according to audio-visual environment characteristics in pedestrian spaces.

## Data availability statement

The datasets presented in this study can be found in online repositories. The names of the repository/repositories and accession number(s) can be found in the article/Supplementary material.

## Author contributions

XR contributed to design the study. XR, PW, MY, and SS implemented the field investigation. PW performed the pre-processing and the statistical analyses of the collected data. QW and WS participated in original draft preparation. XR provided the interpretations of the data and wrote the manuscript with input from PW, QW, WS, MY, SS, DZ, and YX. All authors contributed to the article and approved the submitted version.

## Funding

This study was funded by the National Natural Science Foundation of China (51908102), National Environmental Protection Engineering and Technology Center for Road Traffic Noise Control (F20221019), Philosophy and Social Science Planning Fund of Liaoning Province (L21BGL012), and Economic and Social Development Foundation of Liaoning province (2023lslybkt-029).

## Conflict of interest

The authors declare that the research was conducted in the absence of any commercial or financial relationships that could be construed as a potential conflict of interest.

## Publisher’s note

All claims expressed in this article are solely those of the authors and do not necessarily represent those of their affiliated organizations, or those of the publisher, the editors and the reviewers. Any product that may be evaluated in this article, or claim that may be made by its manufacturer, is not guaranteed or endorsed by the publisher.
