# Supplementary material for: The effects of audio-visual perceptual characteristics on environmental health of pedestrian streets with traffic noise: A case study in Dalian, China
Source: Front Psychol. 2023 Mar 31;14:1122639. doi: 10.3389/fpsyg.2023.1122639 (PMC10102546; doi:10.3389/fpsyg.2023.1122639)

Supplement data 1. Mean(M) and standard deviation(SD) scores for each health evaluation indicator

**Indicators**	**M**	**SD**
Willingness to walk	2.86	1.151
Relaxation	2.98	1.065
Safety	3.11	1.088
Beauty	3.10	1.152
Comprehensive comfort	3.05	1.077

Supplement data 2. Mean(M) and standard deviation(SD) scores for each visual perceptual environment indicator

**Indicators**	**Mean**	**SD**
Building form	-0.17	1.117
Quantity of street greening	0.13	1.127
Type of greenery	0.10	1.162
Facilities	-0.24	1.141
Cleanliness	-0.04	1.117
Width of pedestrian space	0.03	1.124
Sky visibility	0.00	1.112
Spatial scale	0.10	1.148
Interface height variation	-0.02	1.095
Interface concavity variation	-0.06	1.067
Building height along the street	-0.23	1.093
Building distance along the street	-0.33	1.221

Supplement data 3. Mean(M) and standard deviation(SD) scores for each soundscape indicator

**Indicators**	**Mean**	**SD**
Acoustic comfort	2.76	1.003
Subjective loudness	2.70	0.908
Preference	2.80	0.956
Annoyance	2.89	1.056

Supplement data 4. *SPL* of traffic noise at different time frames, where the noise maps of pedestrian streets 1-8 (PM15-17:00) were also shown.

	AM9-11:00			PM15-17:00	
	**Mean**	**SD**		**Mean**	**SD**
1	67.69	2.879	1	66.05	3.057
2	71.23	3.512	2	70.95	3.703
3	62.88	2.156	3	65.43	2.029
4	63.86	2.062	4	65.32	1.357
5	66.22	2.161	5	65.48	2.184
6	64.16	2.928	6	65.05	2.850
7	67.33	4.322	7	67.26	4.459
8	63.17	2.920	8	65.91	3.752

PM15-17:00

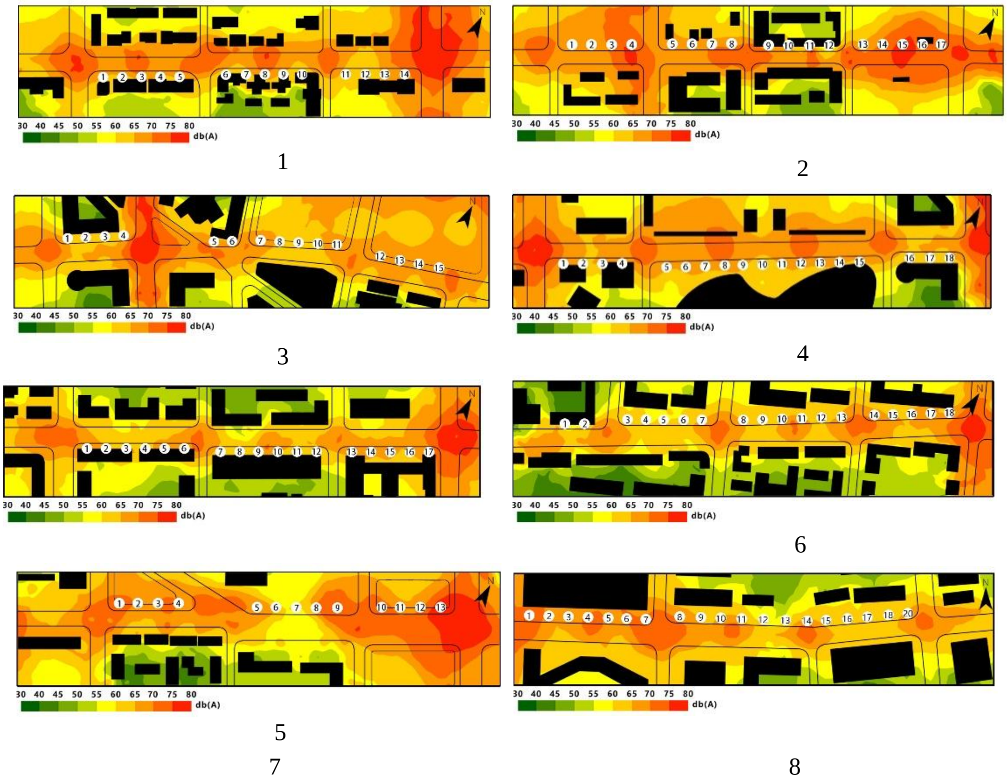

Supplement: Supplementary file 1 [file Data_Sheet_1.PDF]
